# Anesthesia management for cesarean section 10 years after heart transplantation: a case report

**DOI:** 10.1186/s40064-016-2701-8

**Published:** 2016-07-07

**Authors:** Xiaofei Qi, Xiaolei Wang, Xiaolei Huang, Chenhong Wang, Yin Gu, Yuantao Li

**Affiliations:** Department of Anesthesiology, Shenzhen Maternity and Child Healthcare Hospital, Southern Medical University, Shenzhen, 518028 Guangdong China; Department of Anesthesiology, Sun Yat-Sen Cardiovascular Hospital of Shenzhen, Shenzhen, 518028 Guangdong China; Department of Gynecology and Obstetrics, Shenzhen Maternity and Child Healthcare Hospital, Southern Medical University, Shenzhen, 518028 Guangdong China

**Keywords:** Anesthesia, Cardiac transplantation, Cesarean section, Pregnancy

## Abstract

**Introduction:**

Pregnancy after organ transplantation is becoming increasingly common. However, reports of the anesthesia for such patients are rare. Heart transplant recipients are always accompanied with pathophysiological changes and present anesthesiologists with challenge.

**Case description:**

We reported a case of anesthesia management of gravida undergoing cesarean section 10 years after cardiac transplantation. We used two points spinal and epidural anesthesia, combined with phenylephrine throughout the surgery. The course was absolutely successful and both mother and baby got good results.

**Discussion and evaluation:**

Physiology of heart transplant recipients and key points of anesthesia management were discussed.

**Conclusions:**

Spinal anesthesia can be performed in heart transplant recipients, however, we have to think twice before anesthesia for this kind of patients.

## Introduction

For severe end-stage heart disease, cardiac transplantation is a life-saving procedure for those are refractory to medical therapies. Nowadays, the overall survival of recipients has increased to about 90 % at 1 year and more than 75 % at 7 years post transplantation (Taylor et al. 2007). In these heart transplanted recipients, women constitute one-third and about 20 % of them are in reproductive age (Alston et al. 2001).

Cardiac-transplanted patients present anesthesiologists with challenging problems related to the function of the denervated heart and their complex drug therapies. If combined with pregnancy, changes accompanied with pregnancy should be taken into account, and the condition will be more complicated.

We reported the successful outcome of anesthesia for a pregnancy undergoing cesarean section 10 years after cardiac transplantation for a dilated cardiomyopathy. We used intrathecal anesthesia, combined with vasoconstrictor throughout the surgery. The course was uneventful and hemodynamic stable.

## Case description

A 33-year-old pregnant woman was admitted to hospital on 19th, March, 2015 with gestation of 34 weeks and 3 days. She underwent orthotopic cardiac transplantation in September 2005 for a dilated cardiomyopathy. During remaining 10 years she was treated with immunosuppressor tacrolimus and mycophenolate on schedule and no rejection episode was noted. Seven months ago she found she was pregnant and stopped mycophenolate according to the doctor’s advice. During pregnancy, antenatal cares were performed timely and no obstetrical complications were found. After discussion of obstetricians, cardiologists, neonatologists and anesthesiologists, cesarean section was decided to perform on gestation of 35 weeks for her history of heart transplantation.

## Preoperative evaluation

The parturient was 35 weeks gestation and 56 kg on the day of surgery (24th, March, 2015). The patient’s general condition was good and cardiac function classification was stage one. ECG showed sinus tachycardia: 110 beats per minute. Cardiac ultrasound showed left ventricular wall thickening and ascending aortic dilatation. Laboratory tests: Hb 95 g/l. WBC 13.2 × 10^9^/l. Coagulation function, liver and kidney function were normal.

## Anesthesia procedure

The patient fasted overnight and no preoperative medication was administered. Tacrolimus was treated orally 1.5 mg/12 h until morning of surgery. On arrival in the operating room, pulse oxygen saturation, electrocardiogram, and non-invasive blood pressure were monitored, and the baseline values were recorded. Oxygen (5 l/min) by facemask was given until delivery. An intravenous catheter was placed and the patient was preloaded with Lactated Ringer’s Solution (12–15 ml/kg) before induction of spinal anesthesia. Left radial artery was punctured and catheter was inserted to measure direct blood pressure. Deep venous puncture was not performed.

Two points of combined spinal and epidural anesthesia (CSEA) was performed with the patient in the lateral decubitus position. Firstly at L2-3 intervertebral space epidural catheter was placed 3 cm cephalic through epidural needle. Then at L3-4 intervertebral space a 25G spinal Quincke needle was introduced to subarachnoid space, after free flow of cerebral spinal fluid (CSF), 0.5 % ropivacaine 10 mg was injected at a rate of 0.1 ml/s. The patient was immediately placed in the supine position with uterus leftward.

At the same time when anesthesia performed, intravenous phenylephrine was pumping continuously at rate of 0.1 µg/kg.min to prevent hypotension. After injection of intrathecal medication, the rate of phenylephrine was adjusted between 0.1 and 0.3 µg/kg min according to patient’s hemodynamic condition.

When satisfactory anesthesia level (T6) was achieved, surgery began. Five minutes later, a male infant was delivered, weighing 2150 g with Apgar score of 10 at 1 and 5 min after delivery. Immediately after baby was born, oxytocin 10 units was given intramuscular in the uterus and 10 units intravenous dripping with added to 500 ml Lactated Ringer’s Solution. The surgery lasted for 35 min and the course was uneventful, the parturient complained no discomfort. During the surgery, blood pressure maintained at 108–122/65–82mmHg, and heart rate at 80–108 beats/min. The total volume of infusion fluid was 1500 ml, blood loss was 200 ml, urine was 100 ml. When the operation was over, epidural morphine 2 mg was given via epidural catheter and patient controlled intravenous analgesia (PCIA) pump was treated. The pump was total 100 ml contained 1 µg/ml sufentanil with background flow 2 ml/h, bolus 4 ml, locked time 30 min.

She was taken care in intensive care unit postoperaton. 3.5 h later, anesthesia was completely subsided. Tacrolimus was treated orally 1.5 mg/12 h continually. Breast feeding was not allowed for the risk of immunosuppressant to baby. Six days after delivery she got good recovery with no complications and was discharged from hospital together with her infant. Follow up was carried out 5 months and no episode was found.

## Discussion and evaluation

For heart transplanted recipients undergoing non-cardiac surgeries, we should recognize the physiology of the transplanted heart, pharmacologic effects of immunosuppressive medications and complications accompanied by heart transplantation. For pregnant women, obstetrical conditions also should be considered. We should take care of the patients from preoperative period, intraoperative period, and postoperative period.

### Physiology of transplanted heart and preoperative evaluation and preparation

The transplanted heart is denervated. The remaining atrial cuff of recipient is innervated but hemodynamic unimportant. The donor atrium is denervated but is responsible for the electrophysiological responses of the transplanted heart. It retains its intrinsic control mechanisms which include: a normal Frank-Starling effect, normal impulse formation and conductivity, intact ɑ and β receptors responding to circulating catecholamines (Blasco et al. 2009). At rest, the heart rate is faster than normal at about 90–100 beats per minute because lack of vagal tone (Ramakrishna et al. 2009).

Including the function of transplanted heart, we also have to notice the complications following heart transplantation and the influence of anti-rejection drugs in the heart transplanted recipients. Nearly 75 % of post-transplant recipients develop mild to moderate hypertension as a result of immunosuppressor therapy (O’Boyle et al. 2010). Because cardiac responsiveness during exercise is dependent on circulating catecholamines, beta blockers are best avoided after heart transplantation (Blasco et al. 2009). The denervated heart is vulnerable to an accelerated process of coronary atherosclerosis. Allograft coronary atherosclerotic disease is present in 10–20 % of patients 1 year after transplantation and in near 50 % by 5 years (Ng and Cassorla 2007). Even in angiographically normal coronary arteries, coronary luminal narrowing may develop insidiously. The lack of afferent innervation renders episodes of myocardial ischaemia silent in these patients. Therefore, diagnostic ECG is essential in the perioperative period. If coagulation function is abnormal, intravertebral anesthesia should be avoided. Many immune inhibitors, nonstemidial anti-inflammatory drugs are nephrotoxic drugs, so anesthetics that are excreted mainly by renal clearance should be avoided. Immunosuppressant caused infection remains a major cause of death (Aguero et al. 2008; Van de Beek et al. 2008), thus aseptic technique should be paramount. Invasive monitoring techniques and all forms of instrumentation should be handled with sterile manipulation.

Pregnancy is associated with significant hemodynamic demands. Blood volume increases by 40 % and cardiac output by 30 %. The transplanted heart is denervated and so responds to these demands with adaptive mechanisms: an increase in central venous pressure and preload leads to an increase in stroke volume. Circulating catecholamines allow further increases in cardiac output by increasing heart rate and contractility. If pre-pregnancy cardiac function is normal, the transplanted heart is generally able to adjust to these demands (Wu et al. 2007). In reported cases of pregnancies following heart transplantation, outcomes of pregnancy have been good with no recurrence of cardiac dysfunction in the transplanted heart (Armenti et al. 2008; Humphreys et al. 2012; Kalinka et al. 2014). However, the incidence of maternal complications is increased in heart transplant recipients (Miniero et al. 2004; Sibanda et al. 2007). Hypertension is a significant problem both prior to and during pregnancy (Zurbano et al. 2012; Armenti et al. 2004; Coscia et al. 2010) and it requires meticulous control. The incidence of preeclampsia is approximately 20 % (Zurbano et al. 2012; Armenti et al. 2004; Coscia et al. 2010).

### Anesthesia management

Cesarean section is performed in about 30 % of heart transplanted recipients (Cowan et al. 2012; Wielgos et al. 2009). No matter what anesthesia method to perform, we should maintain hemodynamic stable and protect cardiac function, and keep mother and baby safe. Both general and intravertebral anesthesia were successfully performed in heart transplanted patients (Valerio et al. 2014; Allard et al. 2004). But for pregnant women, the better anesthesia choice is intravertebral anesthesia which produces less impact on baby compared with general anesthesia. This patient is with good cardiac function, normal coagulation function and no other serious complications, so spinal anesthesia was performed to prevent impact of general anesthetics to baby. For the post heart transplanted patients, several points we should notice:

Firstly, appropriate anesthesia level must be controlled. For too high anesthesia level will inhibit sympathetic nerve, dilate vessel which is unfavorable for transplanted heart. The same, too low anesthesia level is not enough for the surgery, and pain will increase oxygen consumption of myocardium. We controlled anesthesia level at T6, and got satisfactory effect and hemodynamic stable.

Secondly, appropriate fluid infusion. The normal heart increases its cardiac output via neural stimuli leading to increases in heart rate and contractility (Schwaiblmair et al. 1999), while the denervated heart lacks the ability to respond acutely to hypovolaemia or hypotension with reflex tachycardia, and dependent upon venous return with an initial increase in left ventricular end-diastolic volume (Blasco et al. 2009; Swami et al. 2011), which mediates an increase in stroke volume and ejection fraction by means of the Frank-Starling mechanism. Adequate preload must be ascertained preoperatively and intravascular volume status maintained intraoperatively. However, too much fluid is also not beneficial for denervated heart, for risks to increase heart load and lead to heart failure. We preloaded 12–15 ml/kg Ringer lactate solution before anesthesia to resist vasodilation caused by anesthesia.

Thirdly, vasoconstrictor phenylephrine was treated intravenously to maintain intravascular volume and keep hemodynamic stable. Phenylephrine is α receptor agonist. As intraspinal anesthesia dilates vessel and relatively decrease blood volume, so phenylephrine is helpful to maintain intravascular volume and keep hemodynamic stable, and doesn’t affect myocardial contractivity. The transplanted heart is more sensitive to drugs directly acts on heart such as adrenaline, norepinephrine, isoprensline than those indirectly drugs as ephedrine, metaradrine. The heart rate shows no response to drugs like atropine, neostigmine, phenylephrine, but will respond to isoproterenol, ephedrine, dopamine.

Fourth, antiseptic measures. Maternal infection is of significant concern, although it is relatively rare in practice. It is recommended that all procedures are performed with strict asepsis and antibiotic prophylaxis be used for all operative and instrumental deliveries. We sterilized carefully before spinal anesthesia procedures, and antibiotics were used throughout the surgery. Invasive central venous pressure (CVP) was not used in this case because of the patient’s preoperative stability, minimal surgical risk, and the low possibility of massive fluid infusion.

### Postoperative care

The parturient was taken good care in ICU. Immunosuppressant drugs tacrolimus was continued to use postoperatively as Knight and Morris suggested (Knight and Morris 2007). Analgesia must be good enough to avoid increasing oxygen consumption of myocardium. In addition, intravenous fluids must be well maintained, and urine output monitored.

## Conclusions

For anesthesia in gravidas following heart transplantation, we should recognize the physiology of the transplanted heart, pharmacologic effects of immunosuppressive medications, obstetrical condition of patients. In addition, understand the importance of preload dependence, proper administration of direct vasoactive drugs if needed, and aware infectious risk. Take care of the patient from preoperative period, intraoperative period, and postoperative period. The most important is to make cardiac function normal, hemodynamic stable, enable mother and baby safe.

## Abbreviations

CSEA: combined spinal and epidural anesthesia
CSF: cerebral spinal fluid
PCIA: patient controlled intravenous analgesia
CVP: central venous pressure
ICU: intensive care unit
